# Grazing buffers the effect of climate change on the species diversity of seedlings in an alpine meadow on the Tibetan Plateau

**DOI:** 10.1002/ece3.4799

**Published:** 2018-12-18

**Authors:** Duo‐Bin Wang, Xin‐You Wang, Yuan Wu, Hui‐Long Lin

**Affiliations:** ^1^ State Key Laboratory of Grassland Agro‐ecosystems, Key Laboratory of Grassland Livestock Industry Innovation, Ministry of Agriculture and Rural Affairs, College of Pastoral Agriculture Science and Technology Lanzhou University Lanzhou China

**Keywords:** alpine meadow, asexual recruitment, climate change, grazing, seedlings, sexual recruitment

## Abstract

Climate change predominated by warming over the past decades has affected plant biodiversity, distribution, and ecosystem functioning in alpine grasslands. Yet, little is known about the interactive effect of climate change and grazing on biodiversity and ecosystem functioning. Here, we conducted a vegetation translocation experiment (ten soil‐vegetation blocks were translocated from high‐altitudinal site 3,245 m to low‐altitudinal site 3,045 m) combined with grazing treatment in an alpine meadow on the Tibetan Plateau. The results showed that (a) translocation induced effect of climate change from harsh, high‐altitudinal site to benign, low‐altitudinal site significantly promoted species richness, and density of asexual and sexual seedling, with an increase in the proportion of asexual recruitment to sexual recruitment; (b) grazing decreased the proportion of asexual seedling to sexual recruitment within community, led to a shift in the dominant plant functional groups from graminoids and legumes to forbs; and (c) grazing partly offset the increased species richness of seedling, but not seedling density, induced by climate change. These findings suggest that moderate grazing may buffer the effect of climate change on the plant community composition, and thus, functional role in alpine meadows. Further understanding the influence of climate change on grassland ecosystems needs to consider the non‐additive effect of grazing and climate change to sustainability of grassland services.

## 1 INTRODUCTION

The global average temperature has increased by 0.065°C per decade since 1880 (IPCC, 2013). The unprecedented rapid climate changes directly affect the natural ecosystem, especially in the alpine region (Garcia, Cabeza, Rahbek, & Araújo, 2014; Seddon, Macias‐Fauria, Long, Benz, & Willis, 2016; Baattrup‐Pedersen et al., 2018). Livestock grazing is one of the most common land uses in natural grasslands, but currently, the majority of grasslands are overused and mismanaged (Oldeman, 1992; Schönbach et al., 2011). Previous reports on global climate change or grazing disturbance mostly concentrated on the plant community structures, species composition, soil nutrient dynamics, and aboveground net primary productivity in alpine grasslands (Arft et al., 1999; Danet, Kéfi, Meneses, & Anthelme, 2017; Klein, Harte, & Zhao, 2007; Li, Wu, Zhang, & Du, 2011; Niu, Zhang, Zhao, & Du, 2010; Post & Pedersen, 2008). However, there is still much unknown about the response of plant diversity and ecosystem functioning to the joint effect of climate change and grazing disturbance in the alpine meadow ecosystem.

Seedling recruitment is considered to be one of the most crucial stages in plant life history because it has a significant impact on the diffusion of the plant population, community succession and biodiversity maintenance (Grime, 2001; Kitajima & Fenner, 2000). Additionally, seedling recruitment is the most vulnerable and sensitive stage in plant life history, so it has always been a hot topic in vegetation dynamics (Fenner & Thompson, 2005; Harper, 1977). Seedling recruitment may vary in plant demographic responses to microenvironment heterogeneity (Klein, Harte, & Zhao, 2004). Successful seedling recruitment directly affects plant settlement and species diversity maintenance in different habitats, especially with warming and grazing disturbances in alpine grassland ecosystems (Enright & Miller, 2007; Tilman, 1997; Tolvanen, Schroderus, & Henry, 2001).

Plant recruitment in grasslands by way of sexual or asexual reproduction mainly depends on the species and environmental conditions and plays a critical role in the renewal and succession of the vegetation community (Tilman, 1997). The plant recruitment process refers to the whole growth stage from seed or vegetative organs to standing vegetation. Sexual recruitment has several advantages in genetic variability, seed production, and potential population expansion; however, asexual recruitment can typically maintain the original characteristics of the female parent in the breeding process, which will not generally cause mutations (Nathan & Muller‐Landau, 2000). In the alpine grassland, sexual recruitment is influenced by the seed characteristics, disturbances, and soil. The alpine environmental factors constrain seed production and reduce the growth and survival rates of seedlings due to the short and cold growing season (Chambers, 1995). Asexual reproduction is the dominant regenerative strategy for plants adapting to cold environments, and it is also the most basic feature of plants in alpine grasslands. Alpine plants have a low rate of seedling establishment from seeds; thus, asexual recruitment plays a crucial role in assuring population growth and stability in severely cold areas (Vera, 1997). This is not, of course, to say that sexual reproduction is unimportant; sexual reproduction can allow rare dispersal events with seeds, which is also important for alpine species to extend their distribution areas or to migrate to a new habitat. Sexual and asexual recruitment may have different major contributions to the genomic structure and population colonization (Kleunen, Fischer, & Schmid, 2002).

In alpine meadows, however, seedling recruitment patterns have remained unclear (Chambers, 1995; Forbis, 2003). Before this research, we propose the following hypothesis: (a) seedling density determines species richness (Henry, Stevens, & Carson, 1999); (b) climate change could decrease species richness and increase seedlings density (Arft et al., 1999; Klein et al., 2007; Post & Pedersen, 2008); (c) grazing could cause plant dominant functional groups changed from graminoids with asexual reproduction to forbs with sexual reproduction (Forbis, 2003).

To compare the responses of sexual and asexual recruitment to climate change and grazing disturbance in the region, we studied seedling regeneration in warm and grazed alpine grasslands on the Tibetan Plateau. The objectives of this study were to (a) assess the effects of climate change and grazing disturbance on the diversity and quantification of sexual and asexual recruitment and (b) explore the responses of seedling recruitment of different functional groups to climate change and grazing disturbance in the alpine grassland community. This study may improve our understanding of the natural seedling recruitment and the potential direction of succession under climate change and grazing disturbance in the alpine grasslands of the Tibetan Plateau.

## 2 MATERIALS AND METHODS

### 2.1 Study site

**Figure 1:**
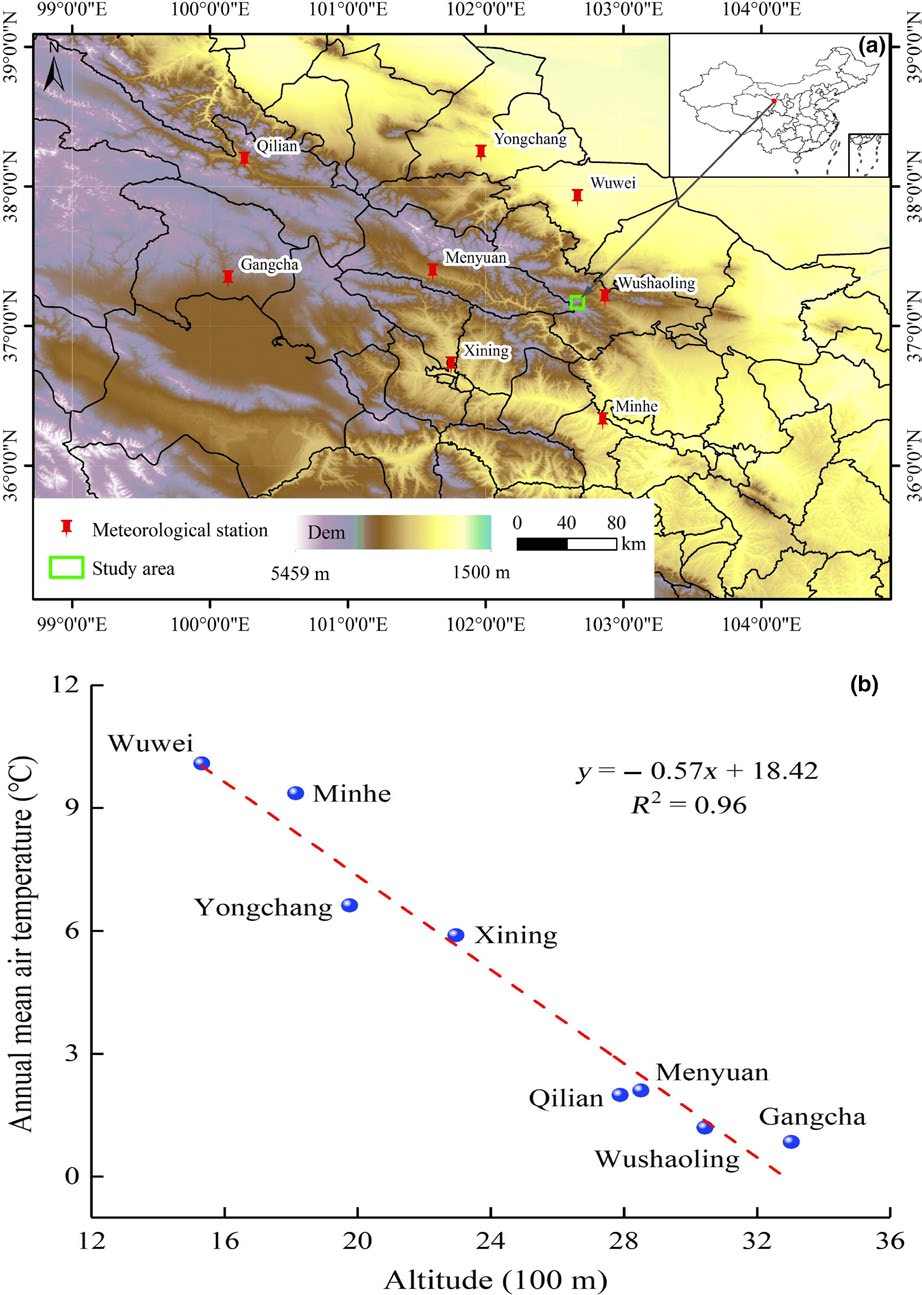
The locations of the study area (Tianzhu alpine pasture, Gansu, China) and surrounding meteorological stations (Qilian, Yongchang, Wuwei, Gangcha, Menyuan, Wushaoling, Xining, Minhe) (a); Linear regression analysis between annual mean air temperature and altitude in the study area (b)

This study was conducted in alpine meadows at 3,000 m a.s.l. in the northeast margin of the Tibetan Plateau in Tianzhu alpine grassland (37°12'N, 102°43'E) in Gansu Province, China (Figure 1a). The average daily air temperature is −0.1°C, ranging from −18.3°C in January to 12.7°C in July. The annual sum of the positive average daily temperature is 1,380°C. The total average annual precipitation is 425 mm, which is concentrated in July, August, and September. This region has a typical continental plateau climate characterized by a long and cold winter, short and mild summer, and no frost‐free period. The plant growth period is 120–140 days. The main soil type of the region is alpine meadow soil, and the vegetation is dominated by *Kobresia humilis, Kobresia capillifolia, Elymus nutans, Polygonum viviparum, Polygonum macrophyllum,* and *Stipa breviflora*. The study area has a history of moderate grazing intensity, and the livestock density is approximately 0.8 heads of yaks per ha.

### 2.2 Air temperatures across altitude

There are eight meteorological stations around the study site, that is, Qilian, Yongchang, Wuwei, Gangcha, Menyuan, Wushaoling, Xining, and Minhe (Figure 1a). We obtained the altitude and average annual temperature at these meteorological stations for 2012, 2013, and 2014 from the National Meteorological Information Center of China (http://data.cma.cn/). Regression analysis detected a negative relationship between altitude and mean annual air temperature, and altitude is the main factor that affects air temperature in the area; the average air temperature elevation gradient is 0.57°C per 100 m (Figure 1b). This finding is consistent with previous reports in the region (Meng et al., 2016; Wang et al., 2014). In this way, the air temperature decreased by approximately 1.14°C from 3,045 m to 3,245 m in the study site.

### 2.3 Controlled warming and grazing experiment

Along a slope, two 50 × 50‐m flat and uniform plots were selected at altitudes of 3,245 m and 3,045 m. Ten randomly selected vegetation‐intact soil blocks (60 × 60 cm, 40 cm deep, including a 50 × 50‐cm quadrat and a 10‐cm‐wide isolation strip) were excavated from the plot at 3,245 m and transferred to the plot at 3,045 m when the soil was slightly frozen in late October 2015. The translocation method caused only minimal damage to the plant roots, as 85% of the root biomass occurred in the upper 10‐cm soil layer (Wang & Shi, 1999). The differences in precipitation, photoperiod and day length were ignored, as the two sites were close to each other (Meng et al., 2016; Wang et al., 2014). A total of ten 50 × 50‐cm quadrats were randomly established in the plot at 3,245 m, five of them were fenced, and the remaining five were used for grazing in 2016. Similarly, ten transferred quadrats were randomly distributed in the plot at 3,045 m, five of them were fenced, and the remaining five were used for grazing. The average distance between the quadrats is about 5 m. In order to prevent the introduction of the surrounding vegetation seeds, we occasionally clipping the vegetation within a 5 m range of the transferred quadrats. Thus, two factors (warming and grazing) resulting four treatments in our study, no warming or grazing (control, C), grazing (G), warming (W), and warming combined with grazing (W × G), respectively. Each treatment had five replicates, and all quadrats were randomly distributed in the two plots.

### 2.4 Seedling demography

Generally, sexual recruitment was produced by seedlings and asexual recruitment by tillers or ramets according to Harper (1977) and Welling & Laine (2002). In early June 2017, seedling species diversity and density were calculated. Seedling species diversity is expressed by the number of species per quadrat, and the seedling density is represented by the plant number per m^2^. All the seedlings of each quadrat were examined for sexual or asexual recruitment, and the sexual recruits were separated from the asexual recruits according to the methods by Wulff (1991): (a) vertical or horizontal direction of the recruits, (b) signs of fragmentation or detachment of the base, and (c) size comparisons. Much attention was paid to differentiate the uniform rounded morphology shown by sexual recruits from the smaller irregular morphology shown by asexual recruits. The classification of plant functional groups according to their ecological characteristics and economic value was as follows: the graminoid functional group included the main species of the Gramineae and Cyperaceae families, the legume functional group included the main species of the Leguminosae family, and the forb functional group included the other species except for those from the Cyperaceae, Gramineae, and Leguminosae families in the community (Tilman 1997; Wright et al., 2005).

### 2.5 Data analysis

In this experiment, the Shapiro‐Wilk test and Levene's test were used for testing residual normality, linearity, and homogeneity of variance. Two‐way ANOVA was performed to determine the statistical significance of the effects of warming and grazing on seedling richness, seedling density, and plant functional groups. Tukey's HSD (Honest Significant Difference) was used for post hoc comparisons. The least significant difference was applied to separate means at the 5% significance level. All data analyses were performed with the SPSS statistical software package for Windows (version 19.0; SPSS Inc., Chicago, IL, USA).

## 3 RESULTS

### 3.1 Species diversity of seedling recruitment

**Figure 2:**
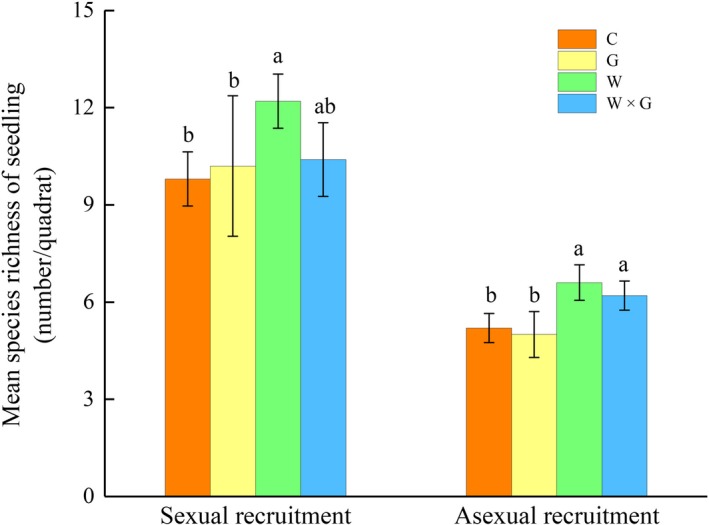
Mean species richness of seedling (±SE) from sexual and asexual recruitment in response to different treatments, no warming or grazing (control, C), grazing (G), warming (W), and warming combined with grazing (W × G), respectively

ANOVA analyses showed that the warming treatment had significantly positive effects on the species richness of seedlings in the alpine meadow (Table 1, Figure 2). Averages of 15, 15.4, 18.8, and 16.6 species were found in C, G, W, and WG per plot (0.25 m^2^), respectively, and in total, 36 seedling species were recorded (Appendix S1). The diversity of the sexual recruitment seedlings was higher than that of asexual (Table 1, Figure 2). Grazing and the interactions between warming and grazing had no significant impact on plant richness. However, grazing reduced the effect of warming on seedling species richness, and sexual recruits accounted for 82% of the reduced species (Figure 2). Therefore, to generalize our findings, warming significantly increased the species diversity of the seedlings in the alpine meadows, while grazing reduced the response of the grasses to warming.

**Table 1:** Summary of two‐way ANOVAs for species richness and density of seedlings using warming and grazing as main factors. Abbreviations are as follows: no warming or grazing (control, C), grazing (G), warming (W), and warming combined with grazing (W × G)

Community characters	Scenario		Source		
			W	G	W × G
	Total	F	13.255	1.961	2.824
		P	0.002	0.181	0.112
Seedling richness	Sexual	F	4.568	1.324	3.27
		P	0.048	0.267	0.089
	Asexual	F	28.167	1.5	0.167
		P	<0.001	0.238	0.689
	Asex/Sex	F	2.736	0.181	1.506
		P	0.118	0.675	0.237
	Total	F	85.364	8.216	12.084
		P	<0.001	0.011	0.003
	Sexual	F	9.765	0.014	11.549
		P	0.007	0.908	0.004
	Asexual	F	115.952	19.309	1.7
		P	<0.001	<0.001	0.211
Seedling density	Asex/Sex	F	12.64	5.056	5.252
		P	0.003	0.039	0.036
	Graminoids	F	128.533	20.291	2.154
		P	<0.001	<0.001	0.162
	Legumes	F	14.389	13.082	0.381
		P	0.002	0.002	0.546
	Forbs	F	6.817	0.495	13.695
		P	0.019	0.492	0.002

### 3.2 Sexual and asexual seedling recruitment

**Figure 3:**
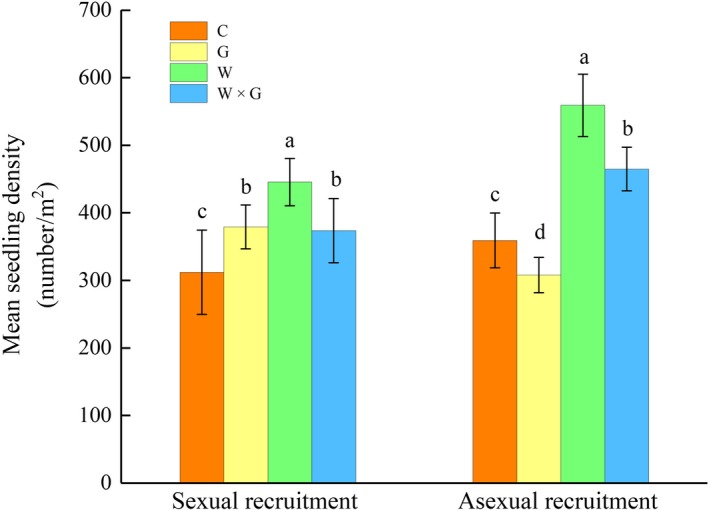
Mean seedling density (±SE) from sexual and asexual recruitment in response to different treatments, no warming or grazing (control, C), grazing (G), warming (W), and warming combined with grazing (W × G), respectively

Overall, warming significantly increased the number of plant seedlings for both sexual and asexual recruitment, and the proportion of asexual recruitment to sexual was highest in the W treatment, followed by WG, C, and G (Table 1, Figure 3). The maximum number of seedlings (1,000.8 m^−2^) was found in the W treatment, followed by WG (838.4 m^−2^), G (687.2 m^−2^), and C (671.2 m^−2^) (Figure 3). Grazing significantly decreased seedling asexual recruitment, and the interaction between warming and grazing significantly increased seedling sexual recruitment (Table 1, Figure 3). Additionally, grazing decreased the proportion of asexual to sexual recruitment in the alpine meadows.

### 3.3 Seedling recruitment by plant functional group

**Figure 4:**
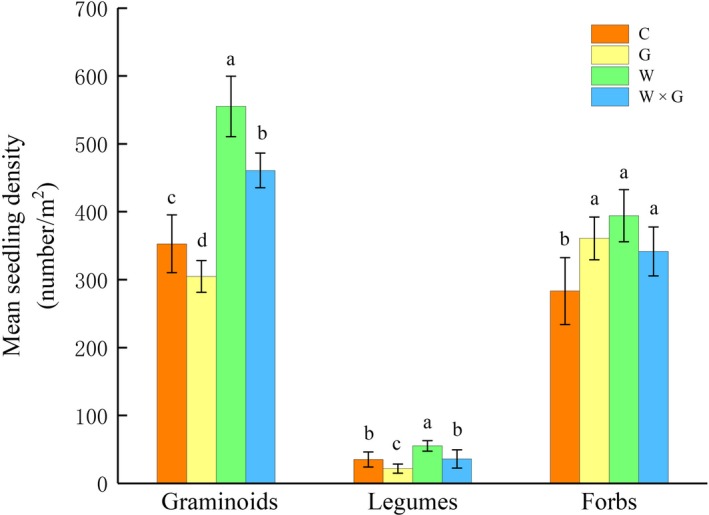
Mean seedling density (±SE) of three plant functional groups in alpine meadows in response to different treatments, no warming or grazing (control, C), grazing (G), warming (W), and warming combined with grazing (W × G), respectively. Plant functional groups: forbs, legumes, and graminoids

Warming significantly increased the seedling number of graminoids, legumes, and forbs (Table 1, Figure 4). Grazing significantly reduced the seedling recruitment of graminoids and legumes but increased forb seedlings. The interaction between warming and grazing significantly increased graminoids and forbs (Table 1, Figure 4).

## 4 DISCUSSION

Global change involves simultaneous changes of multiple driving factors (atmospheric CO_2_ concentration, temperature, ultraviolet radiation, precipitation pattern, etc.), and complex interactions may exist among the factors (Chapin & Shaver, 1996; Terando, Youngsteadt, Meineke, & Prado, 2018). There is no way to completely simulate actual climate change because of the complexity of the ecosystem. Greenhouses, open‐top chambers, soil heating pipes and cables, infrared reflectors, infrared radiators and other warming facilities have both advantages and disadvantages (Niu, Han, Ma, & Wan, 2007). In recent years, increasing attention has been paid to the warming method of soil‐plant transplantation on a small scale along an altitudinal gradient because of the relatively natural temperature gradients for warming (Saleska et al., 2002; Dunne, Saleska, Fischer, & Harte, 2004; Fukami & Wardle 2005; Wang et al., 2014).

A large number of previous studies across alpine or tundra ecosystems indicated that climate change resulted in an approximately 30% species disappearance (Klein et al., 2007; Post & Pedersen, 2008). In our study, however, we found that the seedling species richness and density in the alpine meadow were significantly increased by warming. The results were consistent with a meta‐analysis of 13 study areas in alpine or tundra ecosystems, which showed that warming had positive effects on plant reproduction and growth (Arft et al., 1999). One possible reason is that climate change increases the soil and air temperature, which is a key environmental factor that may promote plant growth, development, and senescence (Wang et al., 2012). In addition, the germination rates of the seeds and organs and soil mineralization increase with increasing temperatures in alpine areas. Seedlings can greatly contribute to the species diversity of grassland communities (Rees, Condit, Crawley, Pacala, & Tilman, 2001). Our study revealed that seedling density did not determine species richness in the alpine meadow dominated by asexual reproduction, and seedling sexual recruitment determines the floristic diversity in alpine meadows. In regard to this result, Parolo and Rossi (2008) thought that sexual spread and reproduction are mostly important for plant colonization at new higher elevation sites; alpine species tend to colonize upwards gradually responding to global climate change mainly through sexual reproduction, which could have a significant influence on the vegetation community structure and species diversity in frigid alpine regions.

Our results indicated that the proportion of asexual to sexual recruitment was the highest in the W treatment, followed by WG, C, and G. This finding was consistent with previous reports that showed that warming increased asexual recruitment but decreased the proportion of forbs in the alpine community (Yahdjian & Sala, 2006). At the same time, different plant species respond differently to temperature. Graminoids may be more sensitive to temperature than forbs and legumes because of their more flexible morphology and greater ability to absorb nutrients (Corazza, Tardella, Ferrari, & Catorci, 2016). Graminoids are generally taller and can provide shade to forbs and legumes in the plant community, which restricts the competition of forbs and legumes for light. Another important reason is root depth; generally, except for a few forbs with taproots, graminoid roots are deeper than those of forbs and legumes, so graminoid plants have higher utilization rates of water and nutrients than forbs and legumes in alpine meadows (Shen, Zhou, Chen, & Zhou, 2002; Wang et al., 2012).

Previous studies showed that plant asexual recruitment was more important in lower temperature areas, that is to say, sexual recruitment plays only a small role in the seedling demographic of an alpine ecosystem (Evette, Bédécarrats, & Bornette, 2009). However, in our study, we found that sexual reproduction increases markedly under grazing conditions. This finding was consistent with previous reports, which indicated that sexual reproduction was very important for seedling recruitment and community regeneration, especially in grazed alpine grasslands (Arft et al., 1999; Forbis, 2003). Grazing influenced the species composition, and the dominant functional groups changed from graminoids with asexual reproduction to forbs with sexual reproduction. Grazing also reduced the number of leguminous seedlings with sexual reproduction (Forbis, 2003). The selective consumption by livestock induces strong changes in the relative abundance of plant species, thereby producing important impacts on ecosystem structure and functioning (Heggenes et al., 2017). As is well known, palatable graminoids and legumes have a more competitive advantage than unpalatable forbs and legumes; thus, grazing may help the seedling recruitment of unpalatable forbs and legumes by decreasing their competition with graminoid species (Gallego, Distel, Camina, & Rodríguez Iglesias, 2004; Niu, He, Zhang, & Lechowicz, 2016). Our study showed that palatable graminoids can be replaced by unpalatable forbs and legumes because yaks and Tibet sheep prefer graminoids over forbs. Animal ingestion also reduces the propagation and availability of palatable graminoids (Moore & Elmendorf, 2006). In addition, grazing provides a favorable amount of sunlight and a greater nutrient availability for seed germination of forbs. The reduction in plant litter is another important mechanism affecting the community structure (Leinaas, Bengtsson, Janion‐Scheepers, & Chown, 2015; Violle, Richarte, & Navas, 2006). Previous study found that plant litter cover may have a negative effect on sexual recruitment (Loydi, Eckstein, Otte, & Donath, 2013). The reduction in sexual recruitment due to plant litter is because plant litter provides a shaded environment to the plant community (Letts, Lamb, Mischkolz, & Romo, 2015). Although litter has positive effects on soil moisture, the importance of soil moisture is limited as a large amount of precipitation is concentrated during seedling recruitment in this area. Therefore, grazing significantly reduced the proportion of asexual recruitment but increased the proportion of sexual recruitment in the alpine community.

## 5 CONCLUSION

In summary, our study added new evidence that global climate change has a positive impact on seedling species richness and density in alpine meadows. The floristic composition and the species diversity are largely determined by sexual seedling recruitment in an alpine community. Grazing changed the species composition and characteristics of the community, and the dominant functional groups changed from graminoids to forbs. Additionally, sexual recruitment plays an important role in community succession and regeneration in alpine grasslands. The findings suggest that global climate change and grazing can affect plant seedling diversity and density in alpine communities and potentially limit their ability to provide essential ecosystem functions and services for human well‐being. Future studies need to continue exploring the dynamics of seedling mechanisms based on monitoring climate change and grazing.

## CONFLICT OF INTEREST

None declared.

## AUTHOR CONTRIBUTION

Huilong Lin and Duobin Wang designed and supervised the project. Duobin Wang, Xinyou Wang, and Yuan Wu conducted the field work and collected the data. Duobin Wang wrote the manuscript with critical input from all the authors.

## Data Availability

Data are available through the Figshare (https://doi.org/10.6084/m9.figshare.7097675).
