# Supplementary material for: Grazing buffers the effect of climate change on the species diversity of seedlings in an alpine meadow on the Tibetan Plateau
Source: Ecol Evol. 2018 Dec 18;9(3):1119–26. doi: 10.1002/ece3.4799 (PMC6374718; doi:10.1002/ece3.4799)
Supplement: Supplementary file 1 [file ECE3-9-1119-s001.docx]

**Appendix 1.** Species list, functional group (FG), family, and palatability of the taxa in the alpine meadow used in our study. G, graminoid, L, legume, F, forb.

| Species identity | FG | Family | Life form | Palatability |
| --- | --- | --- | --- | --- |
| *Allium sikkimense* | F | Alliaceae | Perennial | Palatable |
| *Anaphalislactea* | F | Asteraceae | Perennial | Palatable |
| *Anemone rivularis* | F | Ranunculaceae | Perennial | Unpalatable |
| *Artemisia annua* | F | Asteraceae | Annual | Unpalatable |
| *Artemisia smithii* | F | Asteraceae | Perennial | Palatable |
| *Artemisiamongolica* | F | Asteraceae | Perennial | Palatable |
| *Carexaridula* | G | Cyperaceae | Perennial | Palatable |
| *Delphinium kamaonense* | F | Ranunculaceae | Perennial | Unpalatable |
| *Elymus dahuricus* | G | Gramineae | Perennial | Palatable |
| *Euphorbia altotibetica* | F | Euphorbiaceae | Perennial | Unpalatable |
| *Euphrasia pectinata* | F | Scrophulariaceae | Annual | Unpalatable |
| *Gentianaformosa* | F | Gentianaceae | Perennial | Unpalatable |
| *Gentiana macrophylla* | F | Gentianaceae | Perennial | Palatable |
| *Kobresiacapillifolia* | G | Cyperaceae | Perennial | Palatable |
| *Kobresia humilis* | G | Cyperaceae | Perennial | Palatable |
| *Kobresiapygmaea* | G | Cyperaceae | Perennial | Palatable |
| *Kochia scoparia* | F | Chenopodiaceae | Annual | Palatable |
| *Leontopodiumnanum* | F | Asteraceae | Perennial | Palatable |
| *Medicagoarchiducis-nicolai* | L | Leguminosae | Perennial | Palatable |
| *Notopterygiumincisum* | F | Umbelliferae | Perennial | Palatable |
| *Oxytropislatibracteata* | L | Leguminosae | Perennial | Unpalatable |
| *Pediculariskansuensis* | F | Scrophulariaceae | Annual | Unpalatable |
| *Plantagodepressa* | F | Plantaginaceae | Perennial | Palatable |
| *Poacrymophila* | G | Gramineae | Perennial | Palatable |
| *Polygonum macrophyllum* | F | Polygonaceae | Perennial | Palatable |
| *Polygonum viviparum* | F | Polygonaceae | Perennial | Palatable |
| *Potentilla anserina* | F | Rosaceae | Perennial | Palatable |
| *Potentilla bifurca* | F | Rosaceae | Perennial | Palatable |
| *Potentilla fragarioides* | F | Rosaceae | Perennial | Palatable |
| *Ranunculus tanguticus* | F | Ranunculaceae | Perennial | Unpalatable |
| *SaposhnikoviaSchischk* | F | Umbelliferae | Perennial | Palatable |
| *Saussureasuperba* | F | Asteraceae | Perennial | Palatable |
| *Stellerachamaejasme* | F | Thymelaeaceae | Perennial | Unpalatable |
| *Stipapurpurea* | G | Gramineae | Perennial | Palatable |
| *Taraxacummongolicum* | F | Asteraceae | Perennial | Palatable |
| *Thalictrum alpinum* | F | Ranunculaceae | Perennial | Unpalatable |
